# Phytotoxicity of chitosan-based agronanofungicides in the vegetative growth of oil palm seedling

**DOI:** 10.1371/journal.pone.0231315

**Published:** 2020-04-21

**Authors:** Farhatun Najat Maluin, Mohd Zobir Hussein, Nor Azah Yusof, Sharida Fakurazi, Abu Seman Idris, Nur Hailini Zainol Hilmi, Leona Daniela Jeffery Daim

**Affiliations:** 1 Institute of Advanced Technology, Universiti Putra Malaysia, Serdang, Selangor, Malaysia; 2 Department of Chemistry, Faculty of Science, Universiti Putra Malaysia, Serdang, Selangor, Malaysia; 3 Department of Human Anatomy, Faculty of Medicine and Health Sciences, Universiti Putra Malaysia, Serdang, Selangor, Malaysia; 4 Malaysian Palm Oil Board (MPOB), Kajang, Selangor, Malaysia; 5 Sime Darby Technology Centre Sdn. Bhd., UPM-MTDC Technology Centre III, Universiti Putra Malaysia, Serdang, Selangor, Malaysia; Federal University of Mato Grosso do Sul, BRAZIL

## Abstract

Although fungicides could be the best solution in combating fungal infections in crops, however, the phytotoxic level of fungicides to the crops should be tested first to ensure that it is safe for the crops. Moreover, nanocarrier systems of fungicides could play a significant role in the advancement of crop protection. For this reason, chitosan was chosen in the present study as a nanocarrier for fungicides of hexaconazole and/or dazomet in the development of a new generation of agronanofungicides with a high antifungal potent agent and no phytotoxic effect. Hence, the encapsulation of fungicides into the non-toxic biopolymer, chitosan was aims to reduce the phytotoxic level of fungicides. In the present study, the *in vivo* phytotoxicity of chitosan-fungicides nanoparticles on the physiological and vegetative growth of oil palm seedlings was evaluated in comparison to its pure fungicides as well as the conventional fungicides. The results revealed the formation of chitosan-fungicides nanoparticles could reduce the phytotoxic effect on oil palm seedlings compared to their counterparts, pure fungicides. The chitosan-fungicides nanoparticles were seen to greatly reduce the phytotoxic effect compared to the conventional fungicides with the same active ingredient.

## Introduction

Fungicides are compounds that control the fungal disease by destroying and inhibiting the fungus or fungal spores that cause the disease [1], whereby, the fungal infections on plants may cause a severe decline in crop yield, foliar disease or even cause a severe plant disease [2]. Basal stem rot disease caused by a pathogenic fungus, *Ganoderma boninense* (*G*. *Boninense*) is one of the most serious problems in oil palm cultivation. The fungus causes severe damage to the infected oil palm with a significant loss in the crop yield, hence shorten the productive life of oil palm [3]. Upon infected, the oil palm undergoes wilting and desiccated of frond as well as an unopened spear. The fungus can be spread through the root infection and basidiospores. A healthy palm can be infected by a root contact with the soil inoculum or other infected palm roots [2, 3]

Fungicidal treatments of hexaconazole and dazomet have been proved to inhibit the growth of *G*. *boninense in vitro* antifungal activity study, as well as in infected palm. During the experiment, conventional hexaconazole-based was used as a curative control by applying it in the standing *Ganoderma-*infected palm with the help of a hand-knock injector. The findings show that 74.4% of the treated palm remained alive and produced fruit bunches up to five more years while none from the untreated palm [4, 5]. Hexaconazole has systemic demethylation inhibitors (DMI) that act mainly on the vegetative stage of fungi, by blocking the mycelial growth either inside or on the surface of the host plant [6]. Apart from that, hexaconazole has been widely used as an antifungal agent in crop protection including apple scab, powdery mildew of mango and grape, tikka disease of groundnut, and sheath blight and the blast of rice [7].

Moreover, conventional dazomet-based fumigant was used as a preventive control by eradicating the *Ganoderma* inoculum in the infected palm stump, therefore, minimizing the spread of *Ganoderma* disease within the oil palm plantation [8]. Dazomet is a soil fumigant and degrades toxic gas of methyl isothiocyanate (MITC) when it comes in contact with water or when it breaks down in the soil [9, 10]. MITC is a biocide that acts as an enzyme inhibitor and has been reported in broad-spectrum activities such as inhibit the activity of bacteria, nematodes, and soil-borne pathogenic fungi [11–13]. Apart from that, dazomet has been widely used as soil sterilization in crop disease management [14].

A non-toxic biopolymer of chitosan nanoparticles has been widely used as a carrier of agricultural active ingredient (i.e. pesticides, fertilizer, fungicides, etc.) in crop protection [15, 16]. They exhibit site-specific delivery systems which can solubilize several of the hydrophobic fungicides. Therefore, it enhances the bioavailability and circulation time of the fungicides [17]. Chitosan nanoparticles also can penetrate into the plant tissue, thus allows the efficient delivery of fungicides into the target site of the plant tissue [18]. Apart from that, chitosan is known for its ability to control or reduce the spreading of disease in the plant by inhibiting pathogens and enhance the plant defense mechanism [19, 20].

Nevertheless, it has been argued that the adoption of fungicides tends to affect plant physiology including growth reduction, perturbation of reproductive organ development, carbon metabolism, photosynthesis, and nitrogen alteration despite its ability to control the plant-fungal disease [21–24]. Hexaconazole-induced stress has been reported to have a negative impact on various biological characteristics, including anatomy, physiology, cellular damage, and cytotoxicity of *Pisum sativum* plants [25]. The physiological studies on dazomet have shown the high phytotoxic effect on the oil palm seedlings [26].

Hence, the purpose of the present study was to determine the *in vivo* phytotoxicity effect on the vegetative growth of oil palm seedlings concerning 2 nm of chitosan nanoparticles (CEN), pure hexaconazole, conventional hexaconazole, pure dazomet, conventional dazomet and the three newly-developed systems of chitosan-based agronanofungicides, namely, single-loaded hexaconazole system (chitosan-hexaconazole nanoparticles, CHEN) [27], single-loaded dazomet system (chitosan-dazomet nanoparticles, CDEN) [28], and double-loaded hexaconazole and dazomet system (chitosan-hexaconazole-dazomet nanoparticle, CHDEN) [29]. In each developed system, two different particle sizes were chosen, i.e., 18 nm of CHEN, 168 nm of CHEN, 7 nm of CDEN, 32 nm of CDEN, 5 nm of CHDEN, and 58 nm of CHDEN. The size refers to the mean diameter measured via HRTEM, as previously described [27–29]. In this work, we aim to reduce the toxicological effects of fungicides on the physiological and vegetative growth of oil palm seedlings by the encapsulation of fungicide into the chitosan nanocarrier. This is because chitosan is known for its toxic-free, biodegradability and biocompatibility [30]. Moreover, this work also intended to study the effect of the nanoparticle size on the vegetative growth, physiological and photosynthetic activity of oil palm seedling.

## Materials and methods

### Chitosan-based agronanofungicides and plants materials

CEN (2 nm), CHEN (18 and 168 nm), CDEN (7 and 32 nm), CHDEN (5 and 58 nm) were formulated as previously described [27–29]. Oil palm germinated seeds (10 days old) of commercial *Tenera* (*dura* × *pisifera*) were obtained from the Malaysian Palm Oil Board (MPOB), Kluang. Oil palm germinated seeds (10 days old) of commercial *Tenera (dura* × *pisifera)* were obtained from the Malaysian Palm Oil Board (MPOB), Kluang.

### Experimental design

The nursery trial was conducted at the Malaysian Palm Oil Board (MPOB)’s nursery located at Seksyen 15, Bandar Baru Bangi, Selangor, Malaysia. The experiment was carried out in a randomized complete block design (RCBD) with twelve treatments (Table 1), where H is for hexaconazole, D is for dazomet, and CS is for chitosan, the particle size indicates the mean diameter size measured using HRTEM. Ten germinated seeds were used per treatment with three replications. A total of 360 germinated seeds were used in this experiment. Prior to the treatment, 5 g active ingredient (AI) of treatments (T6–T12) was dissolved in 50 mL of HCl (38% v/v) before top-up to the final volume of 1 L with deionized water. Due to its low water solubility, T2–T4 were dissolved in 10% v/v of ethanol solution. The treatments solution were freshly prepared prior to the applications.

**Table 1:** Treatments of phytotoxicity analysis on germinated seeds of oil palm.

Treatments	Descriptions	Composition (% w/w)	Particle size (nm)	Abbreviation
**T1**	Control, untreated seedling	-	-	-
**T2**	Conventional hexaconazole	H (5)	-	-
**T3**	Pure hexaconazole	H (95)	-	-
**T4**	Conventional dazomet	D (97)	-	-
**T5**	Pure dazomet	D (98)	-	-
**T6**	Chitosan nanoparticles	CS (100)	2	2 nm CEN
**T7**	Chitosan-hexaconazole nanoparticles	CS (85) H (15)	18	18 nm CHEN
**T8**	Chitosan-hexaconazole nanoparticles	CS (83) H (17)	168	168 nm CHEN
**T9**	Chitosan-dazomet nanoparticles	CS (57) D (43)	7	7 nm CDEN
**T10**	Chitosan-dazomet nanoparticles	CS (52) D (48)	32	32 nm CDEN
**T11**	Chitosan-hexaconazole-dazomet nanoparticles	CS (86) H (7) D (7)	5	5 nm CHDEN
**T12**	Chitosan-hexaconazole-dazomet nanoparticles	CS (83) H (8) D (9)	58	58 nm CHDEN

### Experimental conditions

Germinated seeds of oil palm except healthy control were sprayed first with the prepared treatment solution. Then, healthy germinated seeds of oil palm were planted in a soil mixture (topsoil: organic: sand, 3: 2: 1) of polyethylene bag (8 × 12 inches) containing one seed per pot, and watered regularly. Every month, each pot was sprayed (foliar spray) with the treatment solution. The healthy control was germinated seeds, poured with tap water. The phytotoxicity was assessed at 1, 2, and 4 months after the first treatment. The observations included the height of seedling upper the soil, root elongation, dry weight and leaf area at monthly intervals.

### Chlorophyll content and photosynthesis rate

A final observation on chlorophyll content and gas exchange was done at 4 months after sowing. All measurements were carried out between 09:00 until 11:00 with the relative humidity of the air, ambient temperature and ambient CO_2_ concentration were about 70%, 33–36°C and 370–390 μmol.mol^-1^, respectively. The rate of 1000 μmol CO_2_ m^-2^ s^-1^ was used as the light-saturated rate of photosynthesis.

The net photosynthetic rate (*P*_*n*_), stomatal conductance (*G*_*s*_), and concentration of intercellular CO_2_ (*C*_*i*_) were measured on fully expanded leaves seedlings using CIRAS-3 Portable Photosynthesis System (Amesbury, MA, USA). Chlorophyll content was determined with the Chlorophyll Meter, SPAD-502, Konica Minolta (Tokyo, Japan). These measurements were made on five randomly selected seedlings for each treatment with three replicates.

### Statistical analysis

Data are presented as mean ± standard deviation and the statistical difference of the parameters was analyzed using the ANOVA and Tukey’s test (*p* ≤ 0.05) using the SPSS software.

## Results and discussions

**Fig 1:**
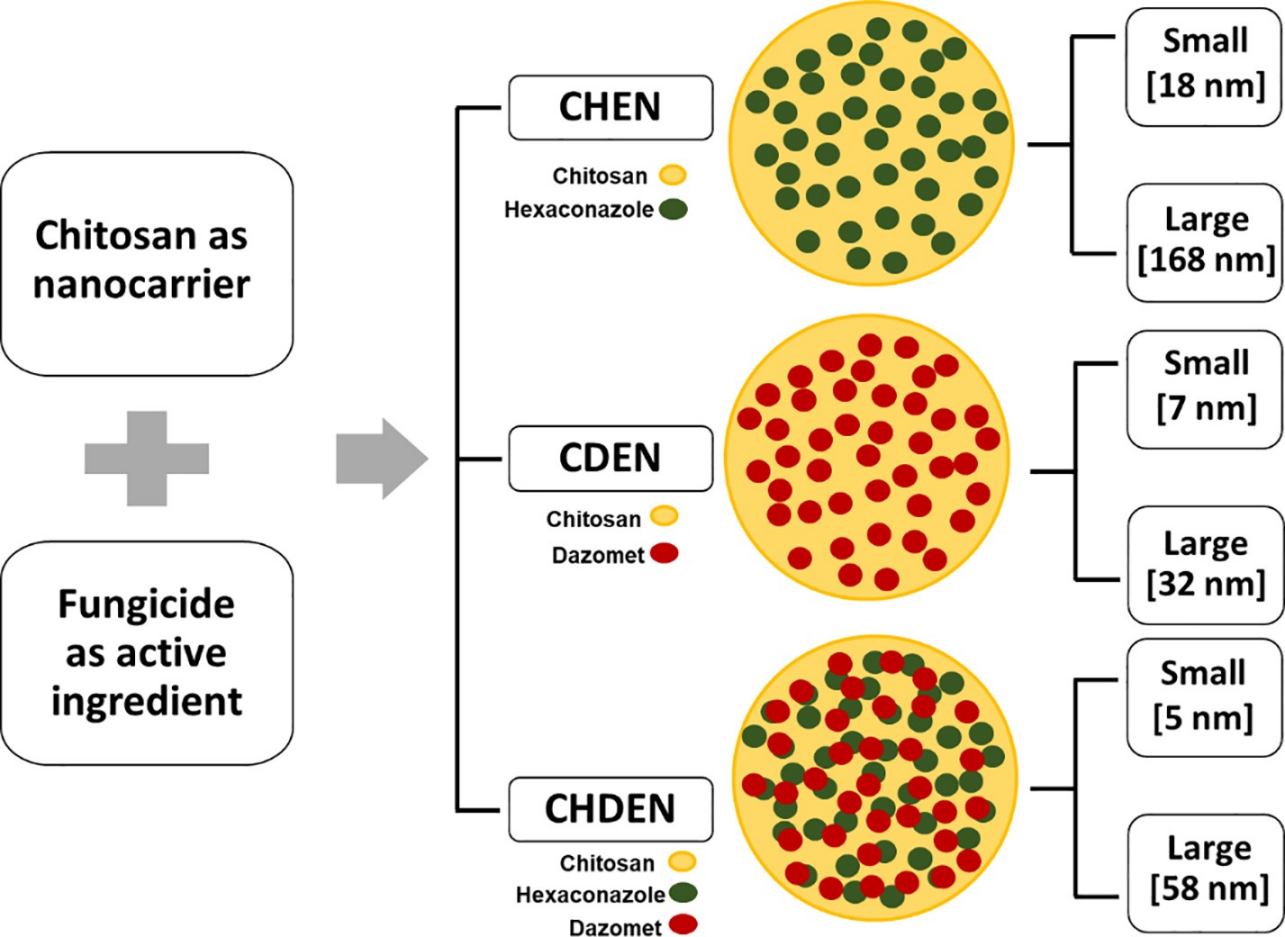
Formation of various chitosan-based agronanofungicides.

The agronanofungicide-based chitosan nanoparticles in this study were prepared in three systems as presented in Fig 1. Chitosan is a nanocarrier, while hexaconazole and/or dazomet are the fungicide active agents. The fungicides were embedded by mixing of hexaconazole and/or dazomet with the chitosan solution. Tween-80 was then added as a stabilizing agent. Then, the crosslinking agent of sodium tripolyphosphate (TPP) was added prior to the formation of the agronanofungicides. As mentioned in previous work, TPP plays an important role in tuning the size of agronanofungicides [27–29]. Hence, for this work, two sizes were chosen in each system, i.e., small and large. The details are given in Fig 1, where CHEN is for chitosan-hexaconazole nanoparticles, CDEN is for chitosan-dazomet nanoparticles, and CHDEN is for chitosan-hexaconazole-dazomet nanoparticle.

### External physiological observation

These experiments were carried out to determine whether the supply of aqueous solution of the treatments influence the vegetative growth of the germinated seeds including seedling height, root elongation, dry weight, leaf area, chlorophyll content as well as photosynthesis rate.

**Fig 2:**
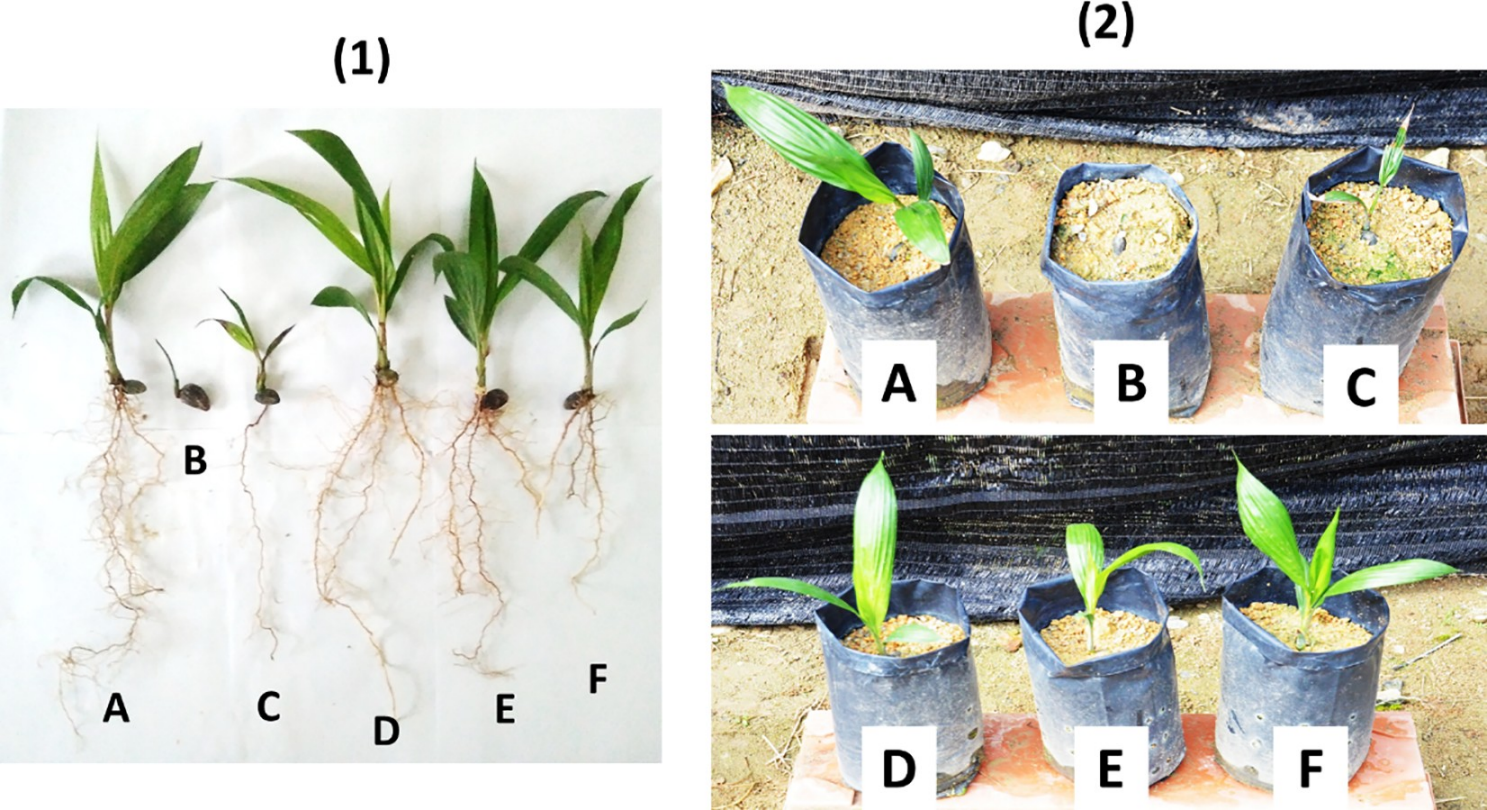
External physiological observation of (1) uprooted and (2) in a polybag of (A) control, (B) conventional hexaconazole, (C) pure hexaconazole, (D) 2 nm CEN, (E) 18 nm CHEN, and (F) 168 nm CHEN of oil palm seedlings at 4 months after sowing.

**Fig 3:**
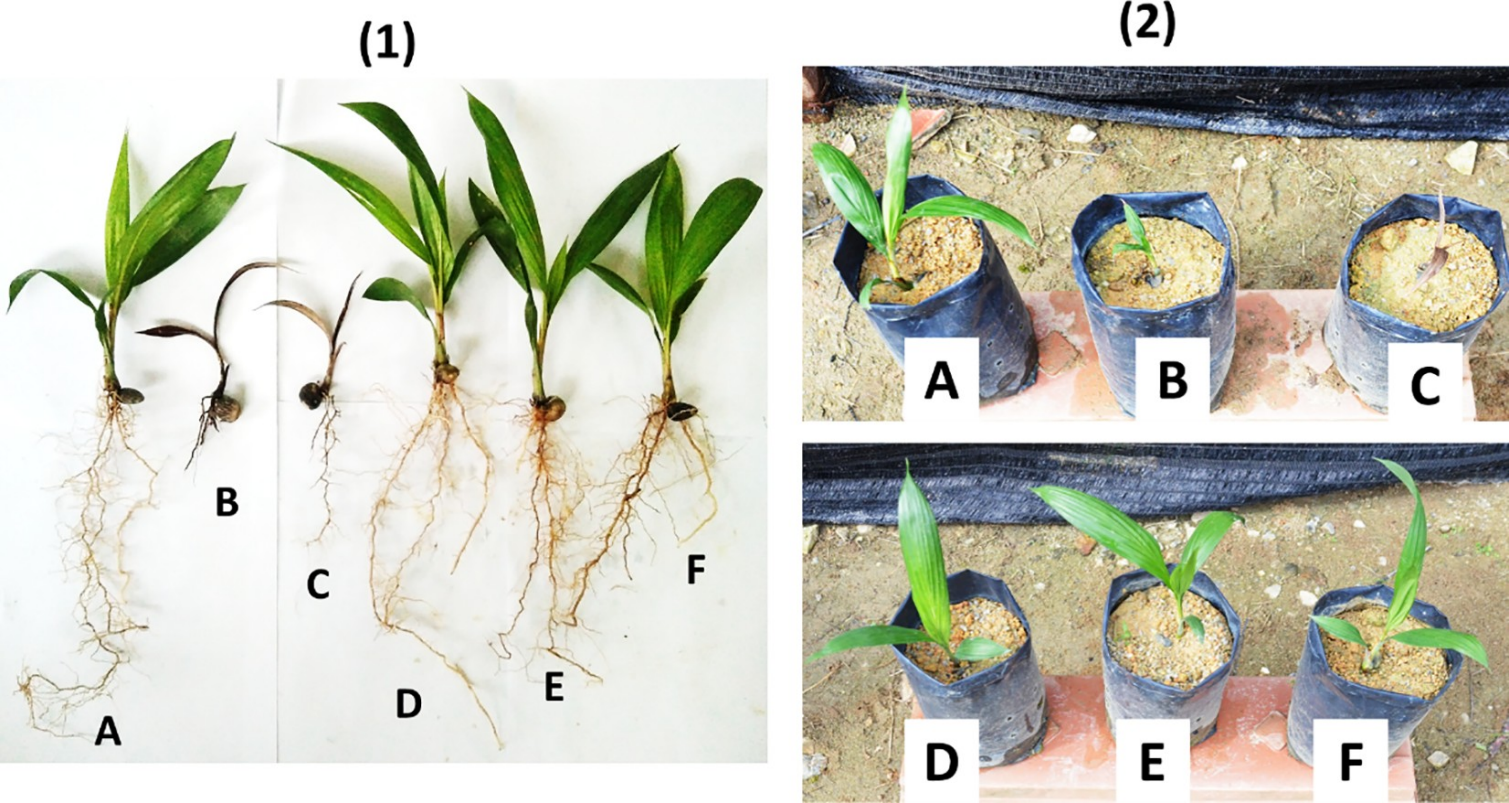
External physiological observation of (1) uprooted and (2) in a polybag of (A) control, (B) conventional dazomet, (C) pure dazomet, (D) 2 nm CEN, (E) 7 nm CDEN, and (F) 32 nm CDEN of oil palm seedlings at 4 months after sowing.

**Fig 4:**
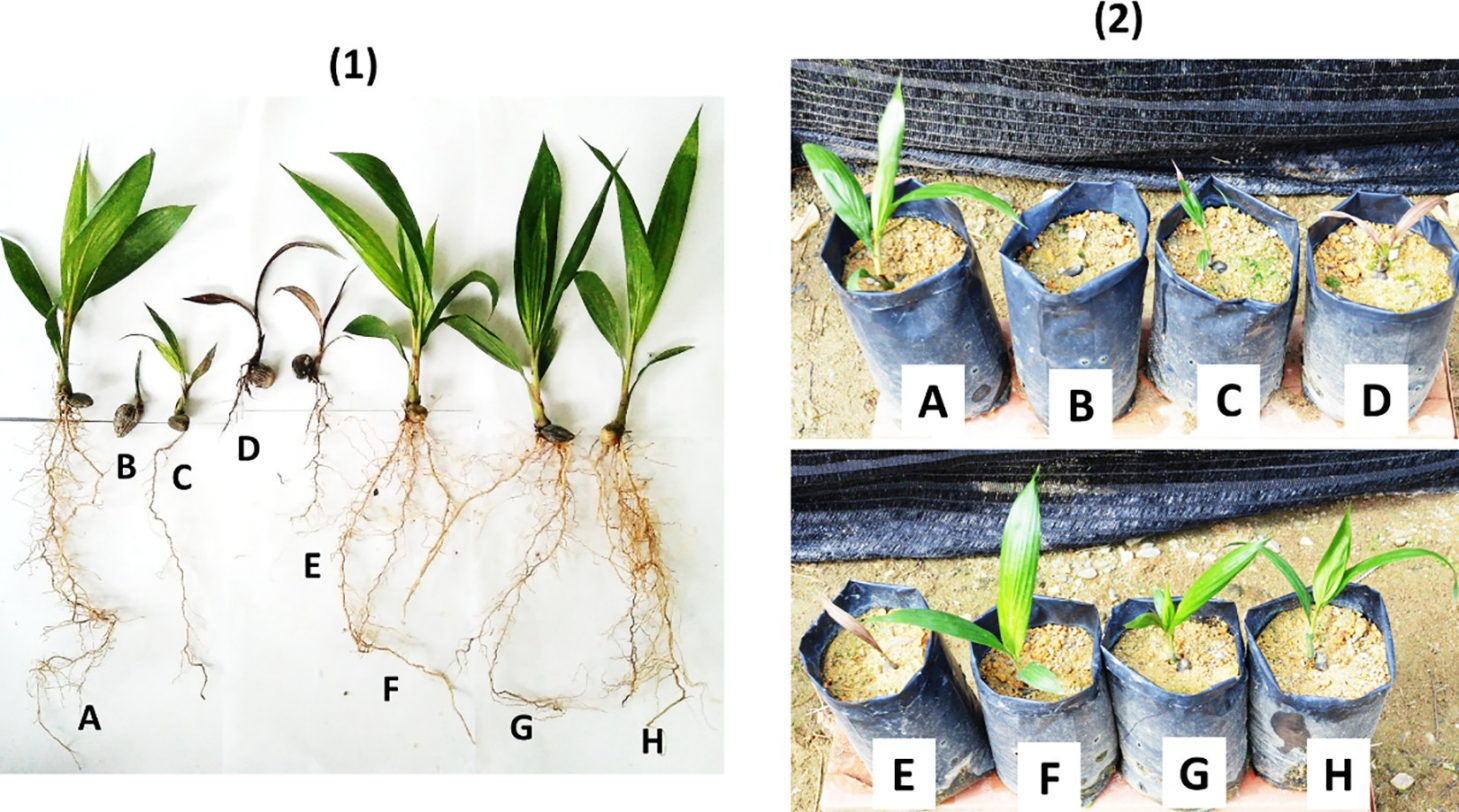
External physiological observation of (1) uprooted and (2) in a polybag of (A) control, (B) conventional hexaconazole, (C) pure hexaconazole, (D) conventional dazomet, (E) pure dazomet, (F) 2 nm CEN, (G) 5 nm CHDEN, and (H) 58 nm CHDEN of oil palm seedlings at 4 months after sowing.

Figs 2 to 4 show the physiological of oil palm seedling uprooted and in polybag at 4 months after sowing. Divided by the three systems, Figs 2, 3 and 4 correspond to the hexaconazole, dazomet and the mixed system of hexaconazole and dazomet, respectively. Fig 2 shows both conventional hexaconazole and pure hexaconazole with a significantly negative influence on the vegetative growth of the oil palm seedlings, where the conventional hexaconazole showed the highest inhibitory effect. On the other hand, the control, 2 nm CEN, 18 nm CHEN and 168 nm CHEN showed either neutral or minimal effect on the vegetative growth of the oil palm seedlings.

Moreover, the application of conventional dazomet and pure dazomet produced a high number of desiccated leaves, which indicated an unhealthy seedling of oil palm (Fig 3). On the other hand, compared to the control, 2 nm CEN, 7 nm CDEN, and 32 nm CDEN showed either neutral or minimal effect on the vegetative growth of oil palm seedlings. Similarly, the double-loaded system of 5 nm CHDEN and 58 nm CHDEN showed the same effects on the vegetative growth of oil palm seedlings, compared to the control (Fig 4).

### Root elongation

Table 2 shows the root elongation of untreated and treated oil palm seedlings with treatments recorded at 0, 1, 2, and 4 months oil palm seedlings after sowing. The results revealed a high phytotoxic effect on root elongation of both pure and conventional of both hexaconazole and dazomet. The seedlings treated with conventional hexaconazole showed the highest inhibitory growth with a complete halt of the root elongation. Meanwhile, pure hexaconazole halted the root elongation after 2 months of sowing, as the length of the root remained the same from months 2 to 4. On the other hand, at 4 months after sowing, conventional dazomet and pure dazomet showed 22% and 53% inhibitory of root growth, respectively, compared to a healthy seedling (control), revealing their high phytotoxic effect on the root. Interestingly, 2 nm CEN, 18 nm CHEN, 168 nm CHEN, 7 nm CDEN, 32 nm CDEN, 5 nm CHDEN, and 58 m CHDEN showed a statistically similar or improved root growth versus a healthy seedling at all months (1, 2, and 4 months after sowing). About 28% and 36% of enhanced root growth were observed in the seedling treated with 2 nm CEN and 58 nm CHDEN at 4 months after sowing. Thus, the results suggested that the incorporation of chitosan in the synthesized CHENs, CDENs, and CHDENs has successfully reduced the negative impact of hexaconazole and dazomet by showing a better vegetative root growth compared to their counterparts, pure fungicides.

**Table 2:** Root elongation (cm) of oil palm seedlings at 0, 1, 2 and 4 months after sowing.

	Months			
Treatments	0	1	2	4
Control, untreated	1.7 ± 0.6^a^	11.4 ± 3.8^a^	20.0 ± 5.9^a^	23.0 ± 6.2^b^
Conventional hexaconazole	1.6 ± 0.7^a^	1.1 ± 0.5^c^	1.0 ± 0.5^c^	1.0 ± 0.5^d^
Pure hexaconazole	1.6 ± 0.6^a^	6.5 ± 2.5^b^	13.4 ± 6.0^b^	13.4 ± 6.0^c^
Conventional dazomet	1.6 ± 0.5^a^	10.2 ± 5.0^ab^	19.7 ± 3.8^a^	18.0 ± 6.5^bc^
Pure dazomet	1.7 ± 0.6^a^	8.9 ± 3.7^ab^	19.8 ± 5.9^a^	10.9 ± 2.0^c^
2 nm CEN	1.7 ± 0.6^a^	12.1 ± 3.2^a^	22.5 ± 4.9^a^	29.5 ± 6.9^a^
18 nm CHEN	1.6 ± 0.8^a^	6.5 ± 2.8^b^	19.9 ± 4.8^a^	26.7 ± 2.6^ab^
168 nm CHEN	1.6 ± 0.6^a^	7.9 ± 3.2^ab^	16.3 ± 6.9^ab^	22.8 ± 3.1^b^
7 nm CDEN	1.7 ± 0.7^a^	12.7 ± 3.7^a^	20.3 ± 7.7^a^	22.4 ± 3.5^b^
32 nm CDEN	1.7 ± 0.6^a^	12.2 ± 4.2^a^	21.5 ± 4.8^a^	24.4 ± 3.8^b^
5 nm CHDEN	1.6 ± 0.7^a^	6.5 ± 3.4^b^	21.9 ± 5.3^a^	25.7 ± 3.3^b^
58 nm CHDEN	1.6 ± 0.8^a^	7.9 ± 3.2^ab^	24.3 ± 5.2^a^	31.3 ± 5.5^a^

Different letters (a, b, c, d) in the same column indicate significant differences between means (*P* ≤ 0.05) according to Tukey’s test.

### Seedling height

Table 3 shows the seedling height of oil palm seedlings untreated and treated with treatments recorded at 0, 1, 2, and 4 months after sowing. The results agreed with the previous results, which revealed a high phytotoxic effect on the seedling height of both pure and conventional of both hexaconazole and dazomet. The seedlings treated with conventional hexaconazole showed the highest inhibitory growth with a complete halt of the seedling height. Meanwhile, pure hexaconazole halted the seedling height after 2 months of sowing, as the length of the shoot remained the same from the months 2 to 4. The conventional and pure dazomet showed 40% and 58% inhibitory shoot growth at 4 months after sowing, respectively, compared to the healthy seedling, thus, revealed their high phytotoxic effect on the shoot. Interestingly, 2 nm CEN, 18 nm CHEN, 168 nm CHEN, 7 nm CDEN, 32 nm CDEN, 5 nm CHDEN, and 58 nm CHDEN showed statistically similar shoot growth compared to the healthy seedling at all months (1, 2, and 4 months after sowing). Therefore, the results suggested that the incorporation of chitosan in the synthesized CHENs, CDENs, and CHDENs were successfully reduced the negative impact of hexaconazole and dazomet by showing a better vegetative shoot growth compared to their counterparts.

**Table 3:** Seedling height (cm) of oil palm seedlings at 0, 1, 2 and 4 months after sowing.

	Months			
Treatments	0	1	2	4
Control, untreated	1.2 ± 0.2^a^	5.9 ± 1.2^a^	14.5 ± 2.8^ab^	26.2 ± 4.4^ab^
Conventional hexaconazole	1.2 ± 0.2^a^	3.3 ± 0.9^b^	2.9 ± 1.5^d^	2.9 ± 1.5^d^
Pure hexaconazole	1.3 ± 0.3^a^	5.1 ± 1.2^ab^	8.3 ± 2.0^c^	8.3 ± 2.0^c^
Conventional dazomet	1.1 ± 0.2^a^	4.0 ± 1.5^ab^	13.6 ± 2.2^b^	15.7 ± 4.0^bc^
Pure dazomet	1.2 ± 0.2^a^	5.3 ± 0.9^a^	13.6 ± 2.2^b^	10.9 ± 3.3^c^
2 nm CEN	1.2 ± 0.2^a^	6.2 ± 2.1^a^	17.1 ± 0.8^a^	29.5 ± 2.4^a^
18 nm CHEN	1.3 ± 0.2^a^	5.2 ± 0.9^a^	11.8 ± 2.8^bc^	19.3 ± 2.6^b^
168 nm CHEN	1.1 ± 0.2^a^	5.1 ± 0.8^a^	11.7 ± 3.2^bc^	19.4 ± 3.1^b^
7 nm CDEN	1.2 ± 0.1^a^	5.8 ± 1.4^a^	14.2 ± 2.0^ab^	22.9 ± 1.5^b^
32 nm CDEN	1.1 ± 0.2^a^	6.6 ± 2.7^a^	14.3 ± 1.6^ab^	24.4 ± 2.9^b^
5 nm CHDEN	1.2 ± 0.2^a^	5.2 ± 0.9^a^	13.3 ± 2.3^b^	26.0 ± 2.3^b^
58 nm CHDEN	1.3 ± 0.1^a^	5.1 ± 0.8^a^	15.7 ± 3.2^ab^	24.2 ± 6.0^b^

Different letters (a, b, c, d) in the same column indicate significant differences between means (*P* ≤ 0.05) according to Tukey’s test.

### Dry weight

The dry weight was measured by drying the uprooted seedlings, which consist of the only shoot and root without the seeds in the oven at 50°C for three days (Table 4). Similar to the previous results, conventional hexaconazole exhibited the highest phytotoxic effect with the lowest dry weight recorded at all months (1, 2, and 4 months after sowing) followed by pure dazomet, conventional dazomet, and pure hexaconazole. Interestingly, 2 nm CEN, 18 nm CHEN, 168 nm CHEN, 7 nm CDEN, 32 nm CDEN, 5 nm CHDEN and 58 nm CHDEN showed statistically similar dry weight compared to the healthy seedlings at 4 months after sowing, indicating zero phytotoxic effect on the dry weight of the oil palm seedling.

**Table 4:** Dry weight (g) of oil palm seedlings at 0, 1, 2 and 4 months after sowing.

	Months			
Treatments	0	1	2	4
Control, untreated	0.02 ± 0.02^a^	0.13 ± 0.03^b^	0.53 ± 0.04^a^	2.31 ± 0.22^a^
Conventional hexaconazole	0.02 ± 0.01^a^	0.08 ± 0.05^b^	0.07 ± 0.02^d^	0.22 ± 0.03^b^
Pure hexaconazole	0.02 ± 0.01^a^	0.10 ± 0.04^b^	0.30 ± 0.09^c^	1.12 ± 0.16^c^
Conventional dazomet	0.02 ± 0.01^a^	0.10 ± 0.05^b^	0.55 ± 0.06^a^	0.69 ± 0.36^c^
Pure dazomet	0.02 ± 0.01^a^	0.22 ± 0.04^a^	0.51 ± 0.02^a^	0.47 ± 0.07^c^
2 nm CEN	0.02 ± 0.01^a^	0.22 ± 0.06^a^	0.52 ± 0.05^a^	2.27 ± 0.22^a^
18 nm CHEN	0.02 ± 0.01^a^	0.16 ± 0.05^ab^	0.47 ± 0.04^ab^	1.75 ± 0.08^ac^
168 nm CHEN	0.02 ± 0.01^a^	0.19 ± 0.09^a^	0.31 ± 0.03^c^	2.22 ± 0.18^a^
7 nm CDEN	0.02 ± 0.01^a^	0.16 ± 0.07^ab^	0.44 ± 0.04^b^	2.24 ± 0.22^a^
32 nm CDEN	0.02 ± 0.01^a^	0.19 ± 0.07^a^	0.55 ± 0.03^a^	2.14 ± 0.25^a^
5 nm CHDEN	0.02 ± 0.01^a^	0.16 ± 0.03^ab^	0.57 ± 0.04^a^	2.41 ± 0.24^a^
58 nm CHDEN	0.02 ± 0.01^a^	0.19 ± 0.05^s^	0.56 ± 0.03^d^	2.26 ± 0.10^a^

Different letters (a, b, c, d) in the same column indicate significant differences between means (*P* ≤ 0.05) according to Tukey’s test.

### Leaf area

Table 5 shows the measured leaf area of oil palm seedlings untreated and treated with various treatments recorded at 0, 1, 2, and 4 months after sowing. Undeveloped leaves were observed in oil palm seedling treated with conventional hexaconazole, as the leaves area remained the same from months 0 to 4. Pure hexaconazole, conventional dazomet, and pure dazomet showed 50%, 65%, and 59% inhibitory on the leaf area, respectively, indicating their phytotoxic effect on the development of leaf. Statistically similar developed leaf area compared to the healthy seedlings were found in the seedlings treated with 2 nm CEN, 18 nm CHEN, 168 nm CHEN, 7 nm CDEN, 32 nm CDEN, 5 nm CHDEN, and 58 nm CHDEN at 2 and 4 months after sowing.

**Table 5:** Leaf area (cm^2^) of oil palm seedlings at 0, 1, 2 and 4 months after sowing.

	Months			
Treatments	0	1	2	4
Control, untreated	0.0 ± 0.0^a^	3.4 ± 0.5^a^	22.7 ± 5.8^a^	52.6 ± 7.6^a^
Conventional hexaconazole	0.0 ± 0.0^a^	0.4 ± 0.1^c^	0.4 ± 0.2^c^	0.4 ± 0.1^c^
Pure hexaconazole	0.0 ± 0.0^a^	3.4 ± 0.4^a^	12.9 ± 5.5^b^	26.2 ± 5.5^b^
Conventional dazomet	0.0 ± 0.0^a^	0.6 ± 0.3^c^	19.2 ± 8.0^ab^	18.4 ± 8.0^b^
Pure dazomet	0.0 ± 0.0^a^	2.0 ± 1.0^b^	18.1 ± 5.0^ab^	21.4 ± 5.0^b^
2 nm CEN	0.0 ± 0.0^a^	3.4 ± 0.3^a^	21.6 ± 2.5^a^	52.6 ± 6.9^a^
18 nm CHEN	0.0 ± 0.0^a^	3.2 ± 0.9^a^	21.3 ± 2.4^a^	53.3 ± 6.7^a^
168 nm CHEN	0.0 ± 0.0^a^	3.5 ± 0.4^a^	20.2 ± 2.9^a^	54.3 ± 6.9^a^
7 nm CDEN	0.0 ± 0.0^a^	2.8 ± 0.9^ab^	19.6 ± 2.0^ab^	49.7 ± 5.4^a^
32 nm CDEN	0.0 ± 0.0^a^	2.5 ± 0.4^b^	18.5 ± 5.1^ab^	50.8 ± 8.4^a^
5 nm CHDEN	0.0 ± 0.0^a^	2.2 ± 0.9^b^	20.1 ± 3.9^a^	48.9 ± 8.7^a^
58 nm CHDEN	0.0 ± 0.0^a^	1.9 ± 0.4^b^	20.5 ± 2.0^a^	49.6 ± 7.4^a^

Different letters (a. b, c, d) in the same column indicate significant differences between means (*P* ≤ 0.05) according to Tukey’s test.

### Leaf desiccation and dead seedling

The percentage of leaf desiccation recorded at 4 months after sowing was calculated based on Eq 1 [26].

$$Leaf\;dessication\;(\%)=\frac{\left[\left(D\times 1)+(Y\times 0.5\right)\right]}{Total\;of\;leaves}\times 100$$ (1)

where 1 is the index for dry/desiccated leaf, and 0.5 is the index for yellowing leaf. A high percentage of desiccated leaves were observed in conventional dazomet and pure dazomet with 85% and 83%, respectively. Moreover, conventional dazomet and pure dazomet also showed a high percentage of dead seedlings with 65% and 71%, respectively, therefore, indicating the high phytotoxic effect of dazomet in oil palm seedlings. In addition, conventional hexaconazole also showed a significant number of desiccations and dead seedlings (14% and 18%, respectively). Interestingly, seedlings treated with pure hexaconazole, 2 nm CEN, 18 nm CHEN, 168 nm CHEN, 7 nm CDEN, 32 nm CDEN, 5 nm CHDEN, and 58 nm CHDEN were perfectly healthy with no desiccated leaf and dead seedling.

### Chlorophyll content and photosynthesis rate

The phytotoxic effect of oil palm seedlings untreated and treated with various treatments on net photosynthesis rate (*P*_*n*_), stomatal conductance (*G*_*s*_), intercellular CO_2_ concentration (*C*_*i*_) and total chlorophyll in leaves of survived oil palm seedlings at 4 months after sowing are presented in Table 6. No data was provided for treatment with conventional hexaconazole due to undeveloped seedling leaf.

**Table 6:** Chlorophyll content (SPAD unit), photosynthesis rate, *P*_*n*_ (μmol CO_2_.m^-2^s^-1^), transpiration rate, *E* (mmol H_2_O.m^-2^s^-1^), stomatal conductance, *G*_*s*_ (μmol.m^-2^s^- 1^) and intercellular CO_2_ concentration, *C*_*i*_ (μmol.m^-2^ s^-1^) of oil palm seedlings treated at 4 months after sowing.

Treatment	Parameters				
	Chlorophyll content	*P*_*n*_	*E*	*G*_*s*_	Intercellular CO_2_ conc.
Control, untreated	43.30 ± 4.75^a^	12.40 ± 1.33^b^	7.55 ± 0.79^b^	370.93 ± 18.84^c^	319.40 ± 14.31^a^
Pure hexaconazole	40.73 ± 4.29^a^	6.37 ± 1.16^c^	6.72 ± 0.41^b^	298.23 ± 20.72^d^	304.80 ± 18.78^a^
Conventional dazomet	26.80 ± 4.31^b^	6.89 ± 2.65^c^	2.61 ± 2.72^c^	213.78 ± 10.46^e^	307.80 ± 43.46^a^
Pure dazomet	29.28 ± 2.72^b^	7.01 ± 2.59^c^	4.85 ± 1.26^c^	188.95 ± 17.78^e^	343.50 ± 56.69^a^
2 nm CEN	43.91 ± 7.01^a^	23.25 ± 1.10^a^	19.68 ± 3.15^a^	731.35 ± 11.54^a^	337.60 ± 20.76^a^
18 nm CHEN	42.52 ± 4.31^a^	21.23 ± 2.92^a^	16.10 ± 2.47^a^	728.07 ± 12.81^a^	323.40 ± 11.06^a^
168 nm CHEN	48.03 ± 5.82^a^	18.16 ± 1.10^ab^	10.98 ± 1.62^b^	490.79 ± 28.53^b^	308.60 ± 9.45^a^
7 nm CDEN	43.92 ± 4.38^a^	21.13 ± 1.36^a^	14.46 ± 3.05^ab^	676.80 ± 36.11^a^	313.80 ± 17.63^a^
32 nm CDEN	45.13 ± 6.03^a^	18.45 ± 0.57^ab^	9.75 ± 2.37^a^	442.78 ± 30.66^b^	297.60 ± 14.55^a^
5 nm CHDEN	47.83 ± 2.56^a^	11.09 ± 1.25^b^	6.02 ± 2.46^b^	265.24 ± 14.02^d^	296.60 ± 14.79^a^
58 nm CHDEN	46.73 ± 4.15^a^	13.32 ±1.84^b^	7.98 ±3.59^b^	359.59 ± 37.76^d^	303.80 ± 10.92^a^

Different letters (a, b, c, d) in the same column indicate significant differences between means (*P* ≤ 0.05) according to Tukey’s test.

The recorded chlorophyll content showed a 38% and 32% decrease compared to the healthy seedlings for conventional dazomet and pure dazomet, respectively. Meanwhile, the other treatments showed no significant difference compared to healthy seedlings. Compared to the healthy seedlings, the *P*_*n*_ value of pure hexaconazole, conventional dazomet, and pure dazomet was decreased by 48%, 44%, and 43%, respectively. In addition, 2 nm CEN, 18 nm CHEN, and 7 nm CDEN showed a remarkable 87%, 71%, and 70% of improved *P*_*n*_ value compared to the healthy seedlings, respectively. The larger size system, 168 nm CHEN and 32 nm CDEN exhibited lower *P*_*n*_ value (compared to their system in a smaller size) but still higher *P*_*n*_ value (46% and 49%, respectively) compared to the healthy seedlings. However, a decrease in the *P*_*n*_ value was observed in the seedling treated with 5 nm CHDEN and 58 nm CHDEN by 10% and 7%, respectively, versus the healthy seedling.

On another note, conventional dazomet and pure dazomet showed the lowest *E* value with 65% and 36% decrease, respectively, compared to the healthy seedlings. Meanwhile, pure hexaconazole showed an 11% decrease in the *E* value compared to the healthy seedlings. Similar to *P*_*n*_ value, 2 nm CEN, 18 nm CHEN, and 7 nm CDEN exhibited a remarkable high *E* value with 160%, 113%, and 92% improvement, respectively, compared to the healthy seedlings. On the other hand, the larger size system, 168 nm CHEN and 32 nm CDEN exhibited lower *E* value (compared to their system in a smaller size) but still showed a 45% and 29% improvement compared to the healthy seedlings, respectively. Moreover, 5 nm CHDEN and 58 nm CHDEN showed the lowest *E* value among the chitosan-based agronanofungicides treatments with statistically similar *E* value to the healthy seedlings.

In addition, *Gs* value of pure hexaconazole, conventional dazomet, and pure dazomet decreased significantly by 19%, 42%, and 49%, respectively, compared to the healthy seedlings. Similar to *Pn* and *E* value, 2 nm CEN, 18 nm CHEN, and 7 nm CDEN exhibited remarkably high *Gs* value with 97%, 96%, and 82% improvement, respectively, versus the healthy seedlings. Moreover, the larger size system, 168 nm CHEN and 32 nm CDEN exhibited lower *Gs* value (compared to their system in a smaller size) but still showed a 32% and 19% improvement, respectively, versus the healthy seedlings. Again, 5 nm CHDEN and 58 nm CHDEN showed the lowest *Gs* value among the chitosan-based agronanofungicides treatments with 28% and 3% decrease, respectively, compared to the healthy seedlings. Moreover, *Ci* value exhibited a negligible significant difference between the healthy seedlings and seedling treated in all treatments.

The results of *P*_*n*_, *E*, and *G*_*s*_ established that 2 nm CEN, 18 nm CHEN and 7 nm CDEN were capable of producing more food by converting the light energy, carbon dioxide and water to carbohydrate (glucose) as shown in Eq 2.

$$6CO_{2}+12H_{2}O+Light\;Energy\to C_{6}H_{12}O_{6}+6O_{2}+6H_{2}O$$ (2)

The effect of particle size can be seen in the *P*_*n*_, *E*, and *G*_*s*_ value, as the larger size system, 168 nm CHEN and 32 nm CDEN produced lower *P*_*n*_, *E*, and *G*_*s*_ value compared to their same system but in a smaller size. This indicated the importance of the size of chitosan-based agronanofungicides in improving the photosynthetic efficiency of oil palm seedling. However, 5 nm CHDEN and 58 nm CHDEN exhibited lower *P*_*n*_, *E*, and *G*_*s*_ value compared to the other chitosan-fungicides nanoparticles system. This may be due to the double-loaded of hexaconazole and dazomet, which then increases the toxic effect on the chlorophyll content and photosynthesis rate of the oil palm seedlings.

The encapsulation of fungicide (hexaconazole and/or dazomet) into the chitosan nanocarrier has significantly reduced the direct phytotoxic effect of the fungicides. This is presumably due to the biocompatibility, biodegradability, and nontoxicity of the natural biopolymer, chitosan [31]. Chitosan is capable of playing a double function as a plant growth enhancer and defense against pathogens [32]. Plants supplied with chitosan nanoparticles have shown capable of enhancing the plant defense response to abiotic and biotic stresses, production of secondary metabolites, and biochemical properties [33].

Moreover, the chitosan encapsulation has been documented to boost fungicide solubility, enhance absorption and site-specific delivery, as well as provide a slow and sustained release [34]. As reported in our earlier work, the release time of hexaconazole encapsulated in CHEN and CHDEN can be up to 86 and 130 hours, respectively, while the release time of dazomet encapsulated in CDEN and CHDEN can be up to 24 and 50 hours, respectively [27–29]. Hence, the slow release properties might also be the reason for the lower phytotoxic effect since the fungicides are gradually released in a sustained manner, instead of fully releasing them at the same time as in the pure fungicides.

Apart from that, no effect of different particle sizes of seedlings treated with the chitosan-based agronanofungicides in the physiological parameter (root elongation, seedling height, dry weight, and leaf area) as well as the percentage of the desiccated leaves and dead seedling. No significant effect was observed in the growth parameter and plant health between the two range sizes of CHENs (18 and 168 nm), CDENs (7 and 32 nm) and CHDENs (5 and 58 nm). This suggested that the growth parameter and plant health were not affected by the particle size of the chitosan-based agronanofungicides. The chitosan nanocarrier itself is enough to shield the phytotoxic effect that comes from the fungicides, therefore showing a high potential of the chitosan-fungicides nanoparticles as the new generation of agronanofungicides with high antifungal efficacy and no phytotoxic effect.

However, the particle size on the single-loaded fungicide of CHENs and CDENs does show an effect on the photosynthetic efficiency in the leaf of the oil palm seedlings, where 2 nm CEN and the smaller size system, 18 nm CHEN and 7 nm CDEN have shown 88%, 71% and 70% improvement in the photosynthesis activity, respectively, compared to the untreated seedlings. This is in agreement with the previously reported work in the supplementation of chitosan nanoparticles in *Robusta* coffee which enhanced their photosynthetic activity and plant growth [35]. This suggested that the increase of photosynthetic activity was due to the penetration of chitosan nanocarrier to the stomata which lead to the increase of the osmosis pressure and consequently lead to the high number of the stomatal cell opening. This is supported by the high number of stomatal conductance of seedlings treated with 2 nm CEN, 18 nm CHEN, and 7 nm CDEN as shown in Table 6.

This is the ideal desired properties for the agronanofungicides for better management of basal stem rot disease of oil palm developed in this work. As previously published, the encapsulation of chitosan enhanced the fungicide solubility and antifungal activity on *Ganoderma boninense*. Moreover, the smaller particle size of the chitosan-based agronanofungicides had shown the higher antifungal activity on *Ganoderma boninense* [27–29]. The half-maximal effective concentration (EC_50_) on *Ganoderma boninense* is 1534.5, 21.4, and 152.2 ng.mL^-1^ for 2 nm of chitosan nanoparticles, hexaconazole, and dazomet, respectively. Moreover, the lower EC_50_ value has been obtained for the chitosan-based agronanofungicides with 8.0, 10.8, 4.6, 20.7, 3.5, and 13.3 ng.mL^-1^ for 18 nm CHEN, 168 nm CHEN, 7 nm CDEN, 32 nm CDEN, 5 nm CHDEN, and 58 nm CHDEN, respectively.

## Conclusion

In summary, the results of the phytotoxic studies revealed the importance of chitosan and its size in reducing the phytotoxicity effect of fungicide in oil palm seedlings. All chitosan-based agronanofungicides have efficiently reduced their phytotoxic impact on oil palm seedlings compared to their counterparts, pure and conventional fungicide. Conventional hexaconazole and dazomet exhibited a strong phytotoxic effect on oil palm seedlings, rendering it harmful to be used in the treatment of fungus of oil palm seedlings. Hexaconazole showed a high impact on the vegetative growth of oil palm seedling, which resulted in growth retardation. On the other hand, dazomet showed an acute phytotoxic effect on oil palm seedlings by showing a high number of desiccated leaves resulting in dead seedlings. On another note, the smaller particle size of chitosan-based agronanofungicides was found to improve the photosynthetic efficiency of the oil palm seedling compared to their same system but in a larger size.

## References

[1] Punja ZK, Utkhede RS. Using fungi and yeasts to manage vegetable crop diseases. Trends Biotechnol. 2003;21(9):400–407. 10.1016/S0167-7799(03)00193-8 12948673

[2] Dias MC. Phytotoxicity: An overview of the physiological responses of plants exposed to fungicides. J. Bot. 2012;2012.

[3] Paterson R. *Ganoderma* disease of oil palm—A white rot perspective necessary for integrated control. Crop Prot. 2007;26(9):1369–1376.

[4] Idris A, Arifurrahman R, Kushairi A. Hexaconazole as a preventive treatment for managing *Ganoderma* in oil palm. MPOB TS Inf. Ser. 2010.

[5] Idris A, Arifurrahman R, Kushairi D. An evaluation of hexaconazole for controlling *Ganoderma* basal stem rot of oil palm in the field as a preventive treatment. PIPOC (Agriculture, Biotechnology and Sustainability) Malaysia 2009.

[6] Khalfallah S, Menkissoglu-Spiroudi U, Constantinidou HA. Dissipation study of the fungicide tetraconazole in greenhouse-grown cucumbers. J. Agric. Food Chem. 1998;46(4):1614–1617.

[7] Thind T. Significant achievements and current status: fungicide research. Plant Pathol. India 2010;267.

[8] Idris A, Maizatul S. Stump treatment with dazomet for controlling *Ganoderma* disease in oil palm. MPOB TS Inf. Ser. 2012;107.

[9] Austerweil M, Steiner B, Gamliel A. Permeation of soil fumigants through agricultural plastic films. Phytoparasitica 2006;34(5):491–501.

[10] Wang D, Fraedrich SW, Juzwik J, Spokas K, Zhang Y, Koskinen WC. Fumigant distribution in forest nursery soils underwater seal and plastic film after application of dazomet, metam‐sodium and chloropicrin. Pest Manag. Sci. 2006;62(3):263–273. 10.1002/ps.1164 16475238

[11] Taylor FI, Kenyon D, Rosser S. Isothiocyanates inhibit fungal pathogens of potato in *in vitro* assays. Plant Soil 2014;382(1):281–289.

[12] Troncoso R, Espinoza C, Sánchez-Estrada A, Tiznado M, García HS. Analysis of the isothiocyanates present in cabbage leaves extracts and their potential application to control *Alternaria rot* in bell peppers. Food Res. Int. 2005;38(6):701–708.

[13] Degenkolb T, Vilcinskas A. Metabolites from nematophagous fungi and nematicidal natural products from fungi as an alternative for biological control. Part I: metabolites from nematophagous *Ascomycetes*. Appl. Microbiol. Biot. 2016;100(9):3799–3812. 10.1007/s00253-015-7233-6 PMC4824826 26715220

[14] Mappes D. In Spectrum of activity of dazomet, IV International Symposium on Soil and Substrate Infestation and Disinfestation 382;1993. p.96-103.

[15] El Hadrami A, Adam LR, El Hadrami I, Daayf F. Chitosan in plant protection. Mar. Drugs 2010;8(4):968–987. 10.3390/md8040968 20479963 PMC2866471

[16] Divya K, Jisha M. Chitosan nanoparticles preparation and applications. Environ. Chem. Lett. 2018;16(1):101–112.

[17] Kashyap PL, Xiang X, Heiden P. Chitosan nanoparticle-based delivery systems for sustainable agriculture. Int. J. Biol. Macromol. 2015;77:36–51. 10.1016/j.ijbiomac.2015.02.039 25748851

[18] Agrawal S, Rathore P. Nanotechnology pros and cons to agriculture: a review. Int. J. Curr. Microbiol. App. Sci. 2014;3(3):43–55.

[19] Ouda SM. Antifungal activity of silver and copper nanoparticles on two plant pathogens, *Alternaria alternata* and *Botrytis cinerea*. Res. J. Microbiol. 2014;9(1):34–42.

[20] Pérez‐de‐Luque A, Rubiales D. Nanotechnology for parasitic plant control. Pest Manag. Sci. 2009;65(5):540–545. 10.1002/ps.1732 19255973

[21] Petit AN, Fontaine F, Vatsa P, Clément C, Vaillant-Gaveau N. Fungicide impacts on photosynthesis in crop plants. Photosynth. Res. 2012;111(3):315–326. 10.1007/s11120-012-9719-8 22302592

[22] Saladin G, Clément C. Physiological side effects of pesticides on non-target plants. Agriculture and Soil Pollution: New Research 2005 p.53–86.

[23] Petit AN, Fontaine F, Clément C, Vaillant-Gaveau N., Photosynthesis limitations of grapevine after treatment with the fungicide fludioxonil. J. Agric. Food Chem. 2008;56(15):6761–6767. 10.1021/jf800919u 18598040

[24] Ney B, Bancal MO, Bancal P, Bingham I, Foulkes J, Gouache D, et al Crop architecture and crop tolerance to fungal diseases and insect herbivory. Mechanisms to limit crop losses. Eur. J. Plant Pathol. 2013;135(3):561–580.

[25] Shahid M, Ahmed B, Zaidi A, Khan MS. Toxicity of fungicides to *Pisum sativum*: a study of oxidative damage, growth suppression, cellular death and morpho-anatomical changes. RSC Adv. 2018;8(67):38483–38498. 10.1039/c8ra03923b PMC9090578 35559088

[26] Mustafa IF, Hussein MZ, Seman IA, Hilmi NHZ, Fakurazi S. Synthesis of dazomet-zinc/aluminum-layered double hydroxide nanocomposite and its phytotoxicity effect on oil palm seed growth. ACS Sustain. Chem. Eng. 2018;6(12):16064–16072.

[27] Maluin FN, Hussein MZ, Yusof NA, Fakurazi S, Idris AS, Hilmi NHZ, et al Preparation of chitosan–hexaconazole nanoparticles as fungicide nanodelivery system for combating *Ganoderma* disease in oil palm. Molecules 2019;24(13):2498. 10.3390/molecules24132498 PMC6651605 31288497

[28] Maluin FN, Hussein MZ, Yusof NA, Fakurazi S, Idris AS, Hilmi NHZ, et al A potent antifungal agent for basal stem rot disease treatment in oil palms based on chitosan-dazomet nanoparticles. Int. J. Mol. Sci. 2019;20(9):2247. 10.3390/ijms20092247 PMC6540247 31067720

[29] Maluin FN, Hussein MZ, Yusof NA, Fakurazi S, Abu Seman I, Zainol Hilmi NH, et al Enhanced fungicidal efficacy on *Ganoderma boninense* by simultaneous co-delivery of hexaconazole and dazomet from their chitosan nanoparticles. RSC Adv. 2019;9(46):27083–27095. 10.1039/c9ra05417k PMC9070574 35528577

[30] Jianglian D, Shaoying Z. Application of chitosan-based coating in fruit and vegetable preservation: a review. J. Food Process. Technol. 2013;4(5):227.

[31] Malerba M, Cerana R. Recent applications of chitin-and chitosan-based polymers in plants. Polymers 2019;11(5):839. 10.3390/polym11050839 PMC6572233 31072059

[32] Hadwiger LA. Multiple effects of chitosan on plant systems: solid science or hype. Plant Sci. 2013;208:42–49. 10.1016/j.plantsci.2013.03.007 23683928

[33] Malerba M, Cerana R. Chitosan effects on plant systems. Int. J. Mol. Sci, 2016;17(7):996. 10.3390/ijms17070996 PMC4964372 27347928

[34] Worrall E, Hamid A, Mody K, Mitter N, Pappu H. Nanotechnology for plant disease management. Agronomy 2018;8(12):285.

[35] Van SN, Minh HD, Anh DN. Study on chitosan nanoparticles on biophysical characteristics and growth of robusta coffee in greenhouse. Biocatal. Agric. Biotechnol. 2013;2(4):289–294.

